# Association of vitamin C intake with breast cancer risk and mortality: a meta-analysis of observational studies

**DOI:** 10.18632/aging.103769

**Published:** 2020-09-29

**Authors:** Dai Zhang, Peng Xu, Yiche Li, Bajin Wei, Si Yang, Yi Zheng, Lijuan Lyu, Yujiao Deng, Zhen Zhai, Na Li, Nan Wang, Jun Lyu, Zhijun Dai

**Affiliations:** 1 Department of Breast Surgery, The First Affiliated Hospital, College of Medicine, Zhejiang University, Hangzhou, China; 2 Department of Oncology, The Second Affiliated Hospital of Xi’an Jiaotong University, Xi’an, China; 3 Breast Center Department, The Fourth Hospital of Hebei Medical University, Hebei Medical University, Shijiazhuang, China; 4 Department of Clinical Research, The First Affiliated Hospital of Jinan University, Guangzhou 510632, China

**Keywords:** vitamin C, breast cancer risk, survival, meta-analysis

## Abstract

The association between vitamin C intake and breast cancer is unclear. This meta-analysis aimed to precisely assess the association of vitamin C intake with breast cancer risk and mortality. We searched the PubMed, Embase, and Web of Science databases up to June 2020 and found 69 studies relevant to breast cancer risk (54 studies) and survival (15 studies). Relative risks and 95% confidence intervals were calculated using the random-effects models. Pooled results suggested that the highest versus lowest vitamin C intake was significantly associated with a lower risk of breast cancer incidence (Relative Risk = 0.86; 95% confidence interval, 0.81–0.92). Dietary vitamin C but not supplements was found to reduce breast cancer risk (Relative Risk = 0.89; 95% confidence interval, 0.82–0.96). For the highest versus lowest vitamin C intake, the pooled hazard risk for breast cancer-specific mortality was 0.78 (95% confidence interval, 0.69–0.88), totality mortality was 0.82 (95% confidence interval, 0.74–0.91), and recurrence was 0.81 (95% confidence interval, 0.67–0.99). Our analysis suggests that higher vitamin C intake is significantly associated with reduced breast cancer incidence and mortality. However, the intake of vitamin C supplements has no significant effect on breast cancer prevention.

## INTRODUCTION

According to the latest study conducted by the International Agency for Research on Cancer, there were 18.1 million new cancer cases and 9.6 million cancer-related deaths globally in 2018 [1]. Based on the latest cancer prediction data, in the USA, the total number of cancer-related deaths in 2019 increased by approximately 4.8% compared with that in 2014 [2]. Breast cancer (BC) is considered the most common cancer and the main cause of cancer-related deaths among women [1]. The global burden of BC in women, therefore, remains large [3]. Thus, it is crucial to prevent BC and improve the long-term survival associated with it. Some previous studies have suggested that lifestyle factors, including intake of fruits, vegetables, fiber, and vitamins, can reduce the risk of BC and improve survival [4–9].

There is a growing interest in vitamin C (VitC) and its health benefits. VitC is hypothesized to reduce the risk of cancer because of its ability to quench free radicals and reduce oxidative damage to DNA [10, 11]. Previous meta-analyses have suggested that high VitC intake can lower colorectal adenoma risk [12]. A dose-response model demonstrated a 14% decrease in gastric adenocarcinoma risk for every 20-mmol/L increase in plasma VitC levels [13]. Another study reported a 15% decrease in endometrial cancer risk for every 50-mg/1000 kcal increase in VitC intake [14]. Similar correlations between VitC intake and reduced cancer risk were observed in lung cancer [15] but not in ovarian cancer [16]. A recent cumulative meta-analysis indicated significant differences in plasma VitC levels of BC patients and control subjects (weighted mean difference = −2.51 μmol/L [95 % confidence interval, −4.00, −1.02, *P* = 0.00]) [17]. To date, increasing epidemiological studies have explored the association between VitC intake and BC risk. Three meta-analyses have reported the association between dietary VitC and BC risk in 1990 [18], 2000 [19], and 2011 [20] and another one on VitC intake and BC survival in 2014 [21]. A recent systematic review found no consistent evidence supporting the anticancer effects of ascorbate when administered to cancer patients orally, intravenously or in combination [5]. However, a study in 2020 reported that high-dose VitC enhanced response to immunotherapy by inducing the infiltration of immune cells into the tumor microenvironment and delaying cancer growth in a T-cell-dependent manner [22]. Therefore, considering the inconsistent findings of previous meta-analyses and reviews, we performed this study to clarify and confirm the correlation between VitC intake and BC-associated risk and survival.

## RESULTS

### Literature selection and study characteristics

**Figure 1:**
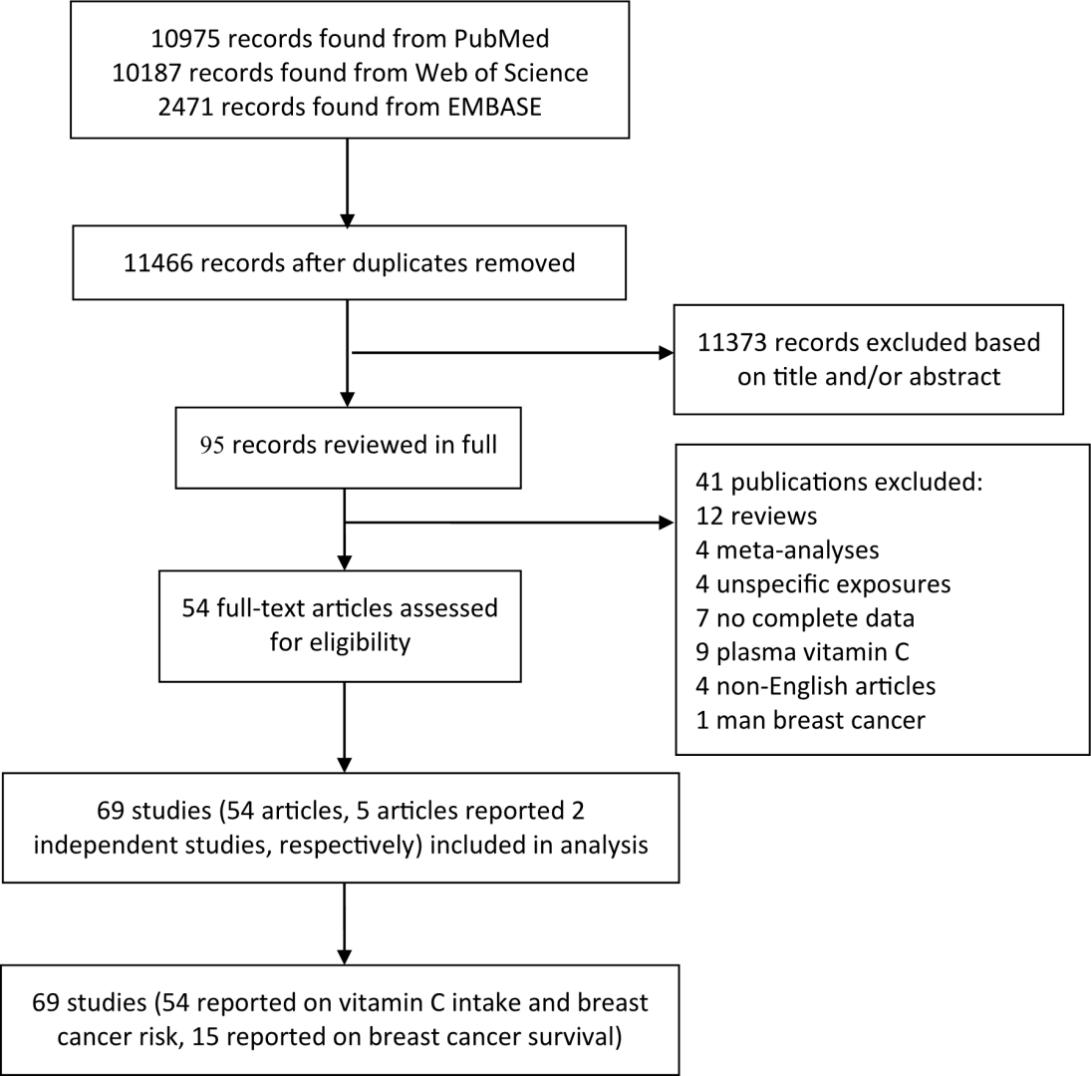
**The precise process of identification and inclusion of studies.**

The flow chart for the selection of publications from the existing literature is shown in Figure 1. In total, 23633 studies were identified by searching the three databases, as well as by manual searching. After the title and abstract review and exclusion of duplicates, finally, 96 articles were reviewed in full. After excluding 39 unqualified articles, we included a total of 54 articles, 44 articles of which (54 studies and 5 articles that reported 2 independent studies) were related to VitC and BC risk, including 24 cohort studies and 30 case-control studies, and 10 articles (15 studies) were selected for analyzing the association between VitC and BC prognosis, including 7, 6, and 2 studies on total mortality, cancer-specific mortality, and recurrence, respectively. Disagreements (such as whether studies that assessed VitC in combination with other nutrients or whether duplicate samples should be included) were resolved after discussion. A third reviewer determined whether or not to exclude these studies to avoid an overestimation of results.

The characteristics of these studies on BC risk published between 1991 and 2016 are presented in Supplementary Table 1. Among them, 24 studies were conducted in America (Canada and the USA), 17 in Europe (France, Netherlands, Denmark, Germany, UK, Switzerland, Greece, Italian, and Spain), and 13 in Asia (Turkey, Korea, China, and Russia). Among 54 studies, 29 provided relative risk estimates for menopausal status, categorized as premenopausal and postmenopausal. Supplementary Table 2 shows the characteristics of the 10 articles on BC prognosis. All included studies reported estimations after adjusting for covariates. Moreover, dietary data in most of the included studies were collected using a validated food frequency questionnaire [23, 24].

### Overall analyses

### VitC intake and BC risk

**Figure 2:**
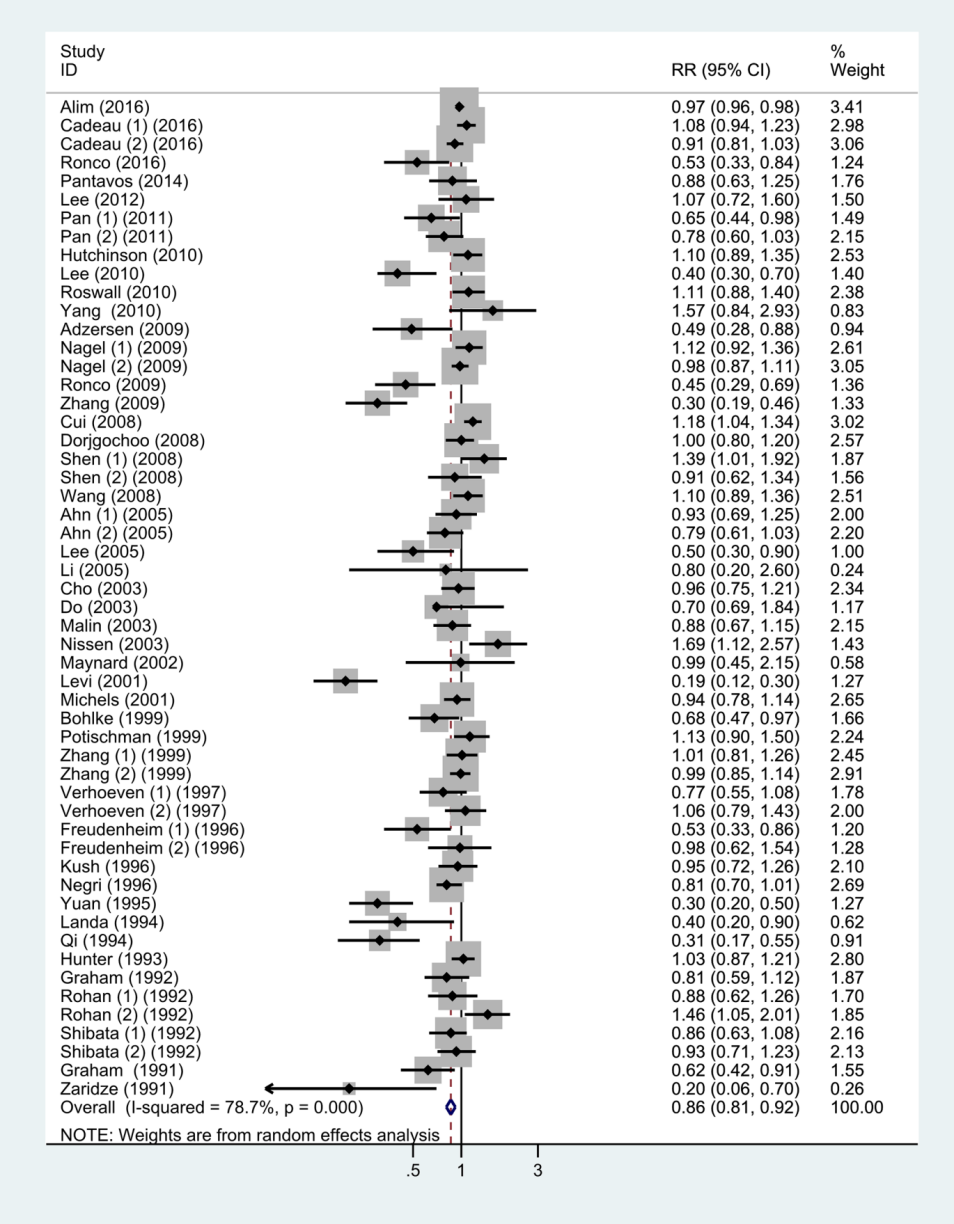
**Forest plot of meta-analysis of breast cancer risk in relation to highest vs lowest categories of vitamin C intake.** Note: Weights are from random-effects analysis. Abbreviations: RR, relative risk; CI, confidence interval.

**Figure 3:**
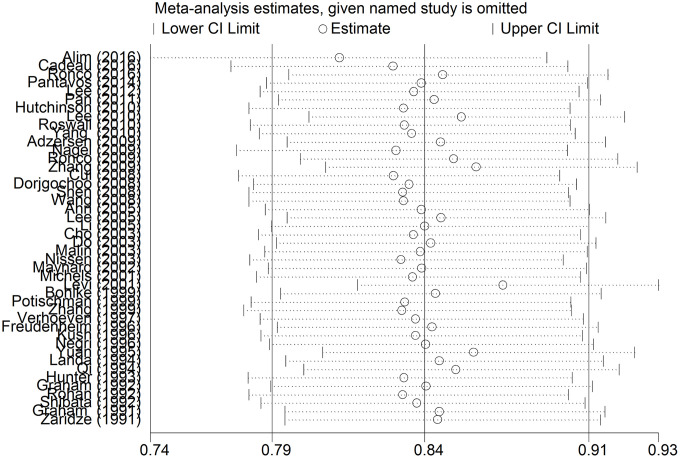
**The sensitivity analysis of the included studies.** Abbreviations: RR, relative risk; CI, confidence interval.

Figure 2 shows the results of the meta-analysis of the association between VitC intake and BC risk. The pooled RR of BC for the highest versus lowest quintile of VitC intake was 0.86 (95% CI = 0.81–0.92; *P* < 0.001) and showed evidence of heterogeneity (*I^2^* = 78.7%, *P* < 0.001). Significant publication bias was observed according to the Begg’s test (P < 0.001) and Egger’s test (P = 0.006). The funnel plot was a little asymmetrical (Supplementary Figure 1). Sensitivity analysis indicated that the overall results remained consistent even after excluding studies individually (Figure 3). The use of the random-effects model indicated that a 100-mg/day increment in VitC intake had no significant effect on BC risk (Supplementary Figure 2). The pooled RRs in the cohort and case-control studies were 1.00 (95% CI = 0.93–1.09, *P* = 0.681, *I^2^* = 40.2%) and 0.86 (95% CI = 0.72–1.03, *P* = 0.107, *I^2^* = 63.1%), respectively.

**Figure 4:**
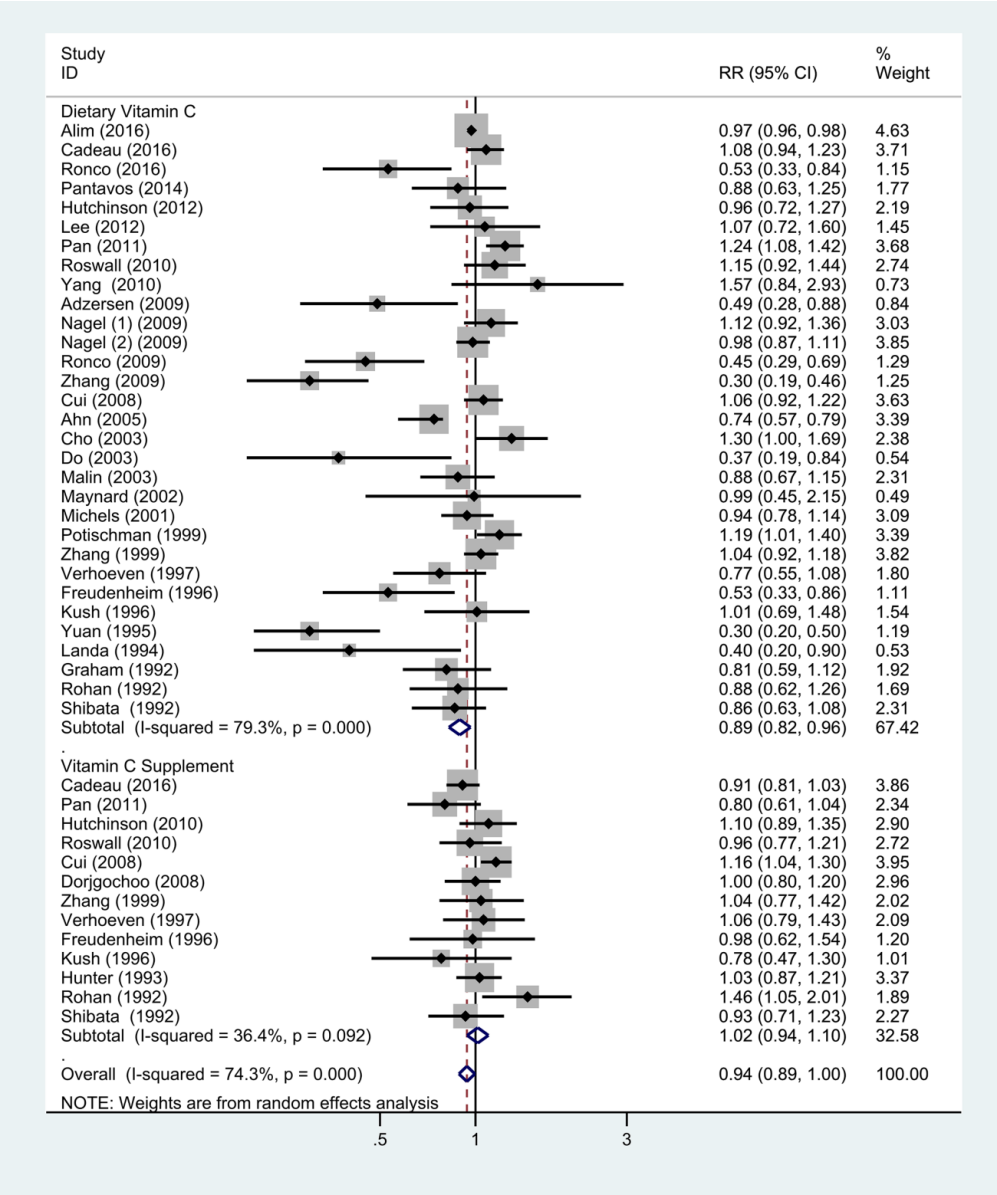
**Subgroup analyses of the associations between breast cancer risk and specific sources of vitamin C.** Note: Weights are from random-effects analysis. Abbreviations: RR, relative risk; CI, confidence interval.

Subgroup analyses were stratified by study design, geographic locations, menopausal status, and source of VitC intake (dietary or supplement). The detailed results are summarized in Table 1. Stratification by study design showed a positive correlation between VitC intake and BC risk in case-control studies (RR = 0.74; 95% CI = 0.65–0.84; *P* < 0.001) and a non-significant inverse association in cohort studies (RR = 0.96; 95% CI = 0.89–1.04; *P* = 0.295) (Supplementary Figure 3). When the studies were stratified by the source of VitC, a significant association was found with dietary intake (RR = 0.89; 95% CI = 0.82–0.96; *P* = 0.004) but not with supplements (RR = 1.02; 95% CI = 0.94–1.10; *P* = 0.678) (Figure 4). In subgroup analyses by geographic location, an inverse association between VitC intake and BC risk was found in Asia (RR = 0.62; 95% CI = 0.48–0.80; *P* < 0.001) but not in Europe and America (Supplementary Figure 4). Furthermore, on stratification by menopausal status, no significant difference was observed among premenopausal (RR = 0.84; 95% CI = 0.72–0.98; *P* = 0.025) and postmenopausal women (RR = 0.88; 95% CI = 0.77–1.00; *P* = 0.045) (Supplementary Figure 5).

**Table 1:** Subgroup analyses of vitamin C intake and breast cancer.

**Analysis specification**	**No. of studies**	**RR(95% CI)**	***P***	**Heterogeneity**	
				**I^2^**	***p***
Highest vs lowest					
All studies	54	0.86 (0.81-0.92)	< 0.001	78.7%	< 0.001
Case-control	30	0.74 (0.65-0.84)	< 0.001	83.9%	< 0.001
Cohort	24	0.96 (0.89-1.04)	0.295	66.0%	< 0.001
Increment of 100 mg/d					
All studies	22	0.94 (0.86-1.03)	0.177	59.1%	< 0.001
Case-control	13	0.86 (0.72-1.03)	0.107	63.1%	0.001
Cohort	9	1.00 (0.93-1.09)	0.681	40.2%	0.100
Menopausal status					
Premenopausal	18	0.84 (0.72-0.98)	0.025	55.1%	0.014
Postmenopausal	11	0.88 (0.77-1.00)	0.045	82.2%	< 0.001
Geographic location					
Europe	17	0.88 (0.77-1.00)	0.054	81.0%	< 0.001
America	24	0.99 (0.93-1.04)	0.062	64.4%	< 0.001
Asia	13	0.62 (0.48-0.80)	< 0.001	88.0%	< 0.001
Source vitamin C					
Dietary	31	0.89 (0.82-0.96)	0.004	79.3%	< 0.001
Supplement	13	1.02 (0.94-1.10)	0.678	36.4%	0.092

Abbreviations: RR, relative risk; CI, confidence interval.

### VitC intake and BC survival

**Figure 5:**
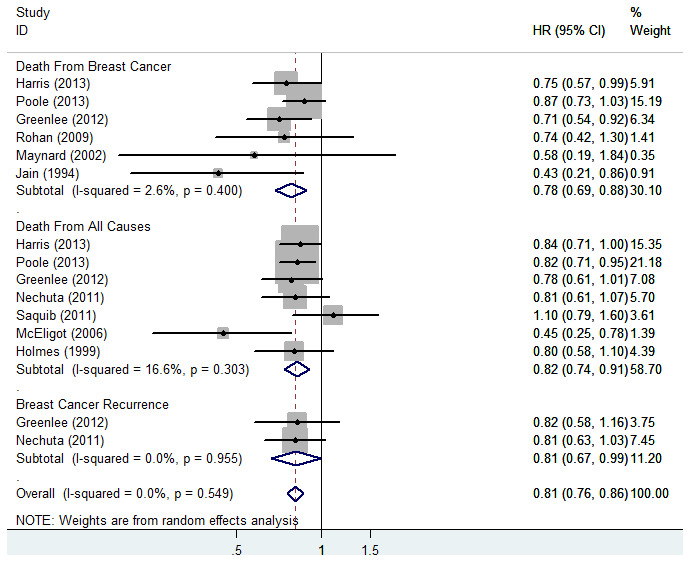
**Forest plot of meta-analysis of breast cancer survival in relation to highest vs lowest categories of vitamin C intake.** Note: Weights are from random-effects analysis. Abbreviations: HR, hazard risk; CI, confidence interval.

While 10 articles (15studies) were selected for analyzing the association of VitC intake with BC prognosis, 7 articles of 10 were chosen to examine its association with total mortality (26347 cases and 3733 deaths) [25–31]. Meanwhile, 3 articles of the 7 also examined BC-specific mortality, two articles of the 7 also examined the risk of BC recurrence (7141 cases and 907 recurrences). Three articles of 10 only evaluated BC-specific mortality [32–34], and all 6 studies assessing BC-specific mortality involved 1513 deaths from 17077 cases. The relevant HRs of VitC intake and risk of death from BC, all causes, and recurrence were calculated using the random-effects model (Figure 5). The pooled HR of BC-specific mortality for the highest versus lowest VitC intake was 0.78 (95% CI = 0.69–0.88; *P* < 0.001) and showed non-significant heterogeneity (*I^2^* = 2.6%) in 6 studies. A significant reduction in the risk of mortality (HR = 0.82; 95% CI = 0.74–0.91; *P* < 0.001, *I^2^* = 16.6%) was observed when comparing the highest versus lowest quintile of VitC intake in 7 studies. For BC recurrence, the combined HR of the 2 relevant studies was 0.81 (95% CI = 0.67–0.99, *P* = 0.043, *I^2^* = 0.0%).

## DISCUSSION

Several studies have assessed the association between antioxidants and the incidence or prognosis of BC [7, 8, 17, 20, 21, 35, 36]. It is well known that VitC is one of the most common antioxidants found in fruits and vegetables with chemo-preventive effects [37]. While two previous meta-analyses have indicated that VitC intake can lower the risk of BC incidence and mortality [20, 21], many other studies, including a randomized clinical trial, have reported no association between either dietary or supplementary VitC intake and BC [38–40]. In contrast, a recent study reported that the use of a VitC supplement increased the risk of BC recurrence and death [41]. Thus, the effect of VitC intake on BC risk and survival remains debatable. This meta-analysis, which focused exclusively on the association between VitC intake and the risks of BC incidence and survival, is therefore significant because it allows a comprehensive understanding and confirmation of this association.

Our meta-analysis included the most up-to-date and comprehensive studies (69 studies) on VitC intake and BC. Previous meta-analyses included other dietary elements (vegetables, fruits, and other vitamins) to assess the risk of BC [19, 20]. However, to the best of our knowledge, this is by far the only meta-analysis to exclusively assess the correlation between VitC intake and BC occurrence and mortality. Our findings indicate that the highest versus lowest VitC intake was significantly associated with a lower BC risk. No significant dose-dependent association was observed between the higher intake of VitC and reduced BC risk. Inverse associations were also found in case-control studies, geographic locations in Asia, and premenopausal women. However, the intake of VitC supplements had no significant protective effects against BC. We also found a lower risk of BC cause-specific mortality, total mortality, and recurrence with the highest VitC intake compared with the lowest intake. Compared with other studies, our study included a larger number of participants, thereby providing more reliable conclusions on the association between VitC intake and BC risk and mortality.

However, there are some inconsistencies between our findings and those of earlier reports. Howe et al. [18] in 1990 reported a significant association between VitC intake and a decrease in the risk for BC in a meta-analysis of 12 case-control studies, which was later confirmed by Gandini et al. [19] in a 2000 meta-analysis that pooled the results of 9 studies. In contrast, a meta-analysis in 2011, including 43 studies, found no significant association between total VitC intake and the risk of BC [20]. After adding the latest relevant studies, our meta-analysis, which included 54 studies, found an inverse association between VitC intake and BC risk when comparing the highest versus lowest quintile of VitC intake.

Several studies have assessed the association between VitC and BC survival; however, the results have been discordant. A previous study found that the risk of recurrence and disease-related mortality were reduced among women taking VitC for more than 3 years [42]. Another meta-analysis suggested that dietary and supplementary VitC intakes were inversely associated with the risk of total mortality and BC-specific mortality [21]. Our meta-analysis showed that the total VitC intake exerted a beneficial effect on reducing the total BC mortality, BC-specific mortality, and recurrence. No previous studies by far have evaluated the association between VitC intake and the risk of BC recurrence. However, a recent Diet, Exercise, Lifestyle, and Cancer Prognosis (DELCaP) study found that the use of VitC supplements was associated with an increased risk of BC recurrence and death [41]. As this is a relatively recent finding, we could not use this data in our analysis. Hence, our assessment is rather conservative given the limited evidence available regarding the association between VitC and BC recurrence. Further large-scale and randomized controlled studies are, therefore, warranted to determine the association between VitC intake and BC survival.

Substantial evidence has supported the beneficial effects of VitC. Because of its antioxidant effects, VitC protects cells from oxidative DNA damage, thus preventing cancer [43, 44]. VitC can effectively remove reactive oxygen metabolites and reactive nitrogen species, including peroxynitrite, nitrogen dioxide, and nitric oxide radicals, thus effectively protecting cellular biopolymers from oxidative damage [45]. Vitamins can also prevent the formation of carcinogenic nitrosamines [46]. It is confirmed that the activation of hypoxia-inducible factors (HIFs) can promote the expression of a stem cell phenotype in BC [47–49]. Ascorbate has recently been reported to inhibit the activation of HIFs, thereby slowing tumor growth [44, 50]. Furthermore, VitC has been shown to cause epigenetic dysregulation, a known driver of malignancy, in tumor and immune cells [22]. It enhances tumor antigenicity and reinforces the functionality of macrophages, natural killer cells, and dendritic cells, thereby enhancing anti-tumor immunity and improving the outcomes of immunotherapy [22, 51–53].

In subgroup analyses, an inverse association was found between VitC intake and BC risk in case-control studies but not in cohort studies, which was in agreement with a previous meta-analysis [20]. This may be due to a larger recall bias in case-control studies for VitC. Furthermore, a significant association was identified between VitC intake and BC in Asia but not in Europe and America. Consistent with a previous study [20], we also found no significant difference in the association between VitC intake and BC both before and after menopause. Separately, another meta-analysis in 1990 [18] reported that the protective effects of VitC were stronger among postmenopausal women than among premenopausal women, although heterogeneity was not significant. Interestingly, a previous meta-analysis found an inverse association between vitamin D intake and BC incidence among premenopausal women, which may be attributed to the anti-cancer mechanisms of vitamin D centering on reproductive hormones and their higher serum levels in premenopausal women [36]. Considering these inconsistencies and without a strong basis for the current results, additional studies exploring the association between VitC intake and BC risk are needed.

Our subgroup analysis also showed an inverse association between dietary VitC and BC. However, this association was not significant for VitC supplement intake in our meta-analysis. These findings are inconsistent with those reported by Hu et al. in 2011 [20], wherein no inverse associations were observed with dietary VitC, but VitC supplementation resulted in a higher risk of BC. However, this association was not relevant as the pooled OR was non-significant (RR = 1.04, 95% CI: 0.94–1.15) in all but one case. Similarly, a single randomized controlled trial showed that the RR was 1.11 (95% CI: 0.87–1.41) in the VitC supplements group [38]. Many molecular studies suggest that VitC can act both as an anti- and pro-oxidant [54]. Some studies have inferred that when the local concentration of VitC is high, it may act as a pro-oxidant and promote oxidative damage to DNA [55–57], whereas the overall evidence thus far indicates no substantial oxidative DNA damage in humans associated with ingesting high amounts of VitC [44, 58, 59]. Nevertheless, the beneficial effects of VitC supplementation in the primary prevention of BC seem to be limited, and additional related research is needed in the future.

Significant heterogeneity was observed in this meta-analysis. Sensitivity analyses suggested that the stable pooled RR was not significantly affected by any single study. We also performed subgroup analyses stratified by confounding factors (study design, geographic locations, menopausal status, and source of VitC intake) to identify the sources of heterogeneity. The consistent results and sensitivity obtained from several subgroup analyses confirm the robustness and reliability of our study.

Like all meta-analysis, this study had potential limitations that need to be acknowledged. First our results showed greater heterogeneity, the source of which remains unknown even after subgroup and sensitivity analyses. This may be attributed to other factors such as different races, different stages of BC, outdoor physical activities, bias in the collection of dietary information, and diverse periods before interview across the included studies. Second, obvious publication bias was admitted, possibly because studies with negative results are more difficult to be published. Third, we did not perform a stratified analysis by hormone status because limited information was available and additional studies are needed. Finally, the interactions between VitC and other vitamins may reinforce the associations with BC, but our estimates of further potential interactions are limited by the lack of raw data from related studies.

In conclusion, the results from this meta-analysis suggest that a high intake of total VitC appears to be significantly correlated with a reduced risk of BC incidence, mortality and recurrence. However, additional VitC supplementation should be cautiously considered for BC prevention.

## MATERIALS AND METHODS

### Literature retrieval

We searched PubMed, Web of Science, and Embase databases for relevant English publications up to June 2020. The terms “breast cancer(s)” [Title/Abstract], “breast tumor(s)” [Title/Abstract], “mammary cancer(s)” [Title/Abstract] and “breast neoplasm(s)” [Title/Abstract] in combination with “Ascorbic Acid” [Mesh], “ascorbic acid” [Title/Abstract], “Vitamin C” [Title/Abstract], “Vitamin C supplement(s)” [Title/Abstract], “antioxidant(s)” [Title/Abstract], “Vitamin(s)” [Title/Abstract] were used in the search. We also identified additional relevant studies by scanning the reference lists of all eligible articles and reviews. Only full-length original articles published in English were included. Two researchers independently read the retrieved documents, screened relevant publications based on the exclusion criteria, and deleted all duplicate studies. Any disagreement between the two authors was resolved by discussion.

### Selection criteria

The inclusion criteria for publications were (1) an original article, (2) a cohort or case-control study in design, (3) the exposure of interest was the use of dietary or supplementary VitC (ascorbic acid), (4) the dependent variable of interest was BC, (5) relative risk (RR), hazard risk (HR), or odds ratio (OR) with a 95% CI was provided, and (6) the results were adjusted at least for age. The exclusion criteria were as follows: (1) case reports, reviews, animal studies, and in-vitro studies; (2) studies reporting insufficient statistics or results; (3) repeated or overlapping publications; (4) studies that assessed VitC in combination with other nutrients; (5) studies assessing the association for blood levels of VitC; and (6) studies in which low/no intake of VitC was not the reference category.

### Data extraction and quality assessment

Data on the study population characteristics, including author names, year of publication, geographic locations, study design, age, assessment of diet and supplement type, duration of follow-up, sample size, and RR (95% CI) for each category of VitC and confounding factors that were adjusted, were independently extracted by two investigators. From each study, we extracted the RR with the most adjusted potential confounders. If the two researchers disagreed regarding the eligibility of data, a consensus was reached with the help of a third reviewer.

Two authors independently assessed the quality of each included study based on the Newcastle-Ottawa Quality Assessment Scale [60]. The content of the study was evaluated for four major aspects: selection, comparability, exposure, and results. Thereafter, the studies were categorized as high, medium, and low quality. A study with a score >6 was considered of good quality.

### Statistical analysis

The pooled measure was calculated as the inverse variance-weighted mean of the logarithm of RR with 95% CI to assess the association between VitC intake and BC. A random-effects model was used to combine the study-specific RR (95% CI), which considered both within-study and between-study variations [61, 62]. Heterogeneity across the included studies was assessed using the Q and I^2^ statistics [63] and was considered significant at *P* values <0.05 in the Q statistic or at I^2^ values ≥50%. We performed subgroup analyses based on menopausal status, study type, geographical location, and VitC source to explore potential origins of heterogeneity. Sensitivity analyses were performed to assess the effect of individual studies on the results [64]. Egger’s test and Begg’s test were used to assess the potential publication bias [65].

For the dose-response analysis of results across the different categories of VitC intake, methods reported by Greenland [66] and Orsini [67] were used to calculate the study-specific slopes (linear trends). We used a two-stage hierarchical regression model to examine the possible linear dose-response association between VitC intake and BC risk [68]. We analyzed the data using the random-effects restricted cubic spline and four knots models. In all studies, the median or mean level of VitC in each category was assigned to the corresponding RR with 95% CI. In studies where VitC was reported as a range of intake, the midpoint of the range was used. For the upper open interval, we assumed that the width of the interval is the same as the width of the adjacent interval. For the lower open interval, we set the lower boundary to zero [15]. The dose-response results in forest plots are presented for every 100-mg/day increment in VitC intake. All meta-analyses were performed using STATA statistical software (version 14.0; StataCorp, College Station, TX, USA). All statistical tests were two-sided, and P values <0.05 were considered significant.

## Supplementary Material

Supplementary Figures

Supplementary Table 1

Supplementary Table 2
