# Supplementary material for: Association of vitamin C intake with breast cancer risk and mortality: a meta-analysis of observational studies
Source: Aging (Albany NY). 2020 Sep 29;12(18):18415–35. doi: 10.18632/aging.103769 (PMC7585084; doi:10.18632/aging.103769)
Supplement: Supplementary Figures [file aging-12-103769-s003..pdf]

SUPPLEMENTARY FIGURES

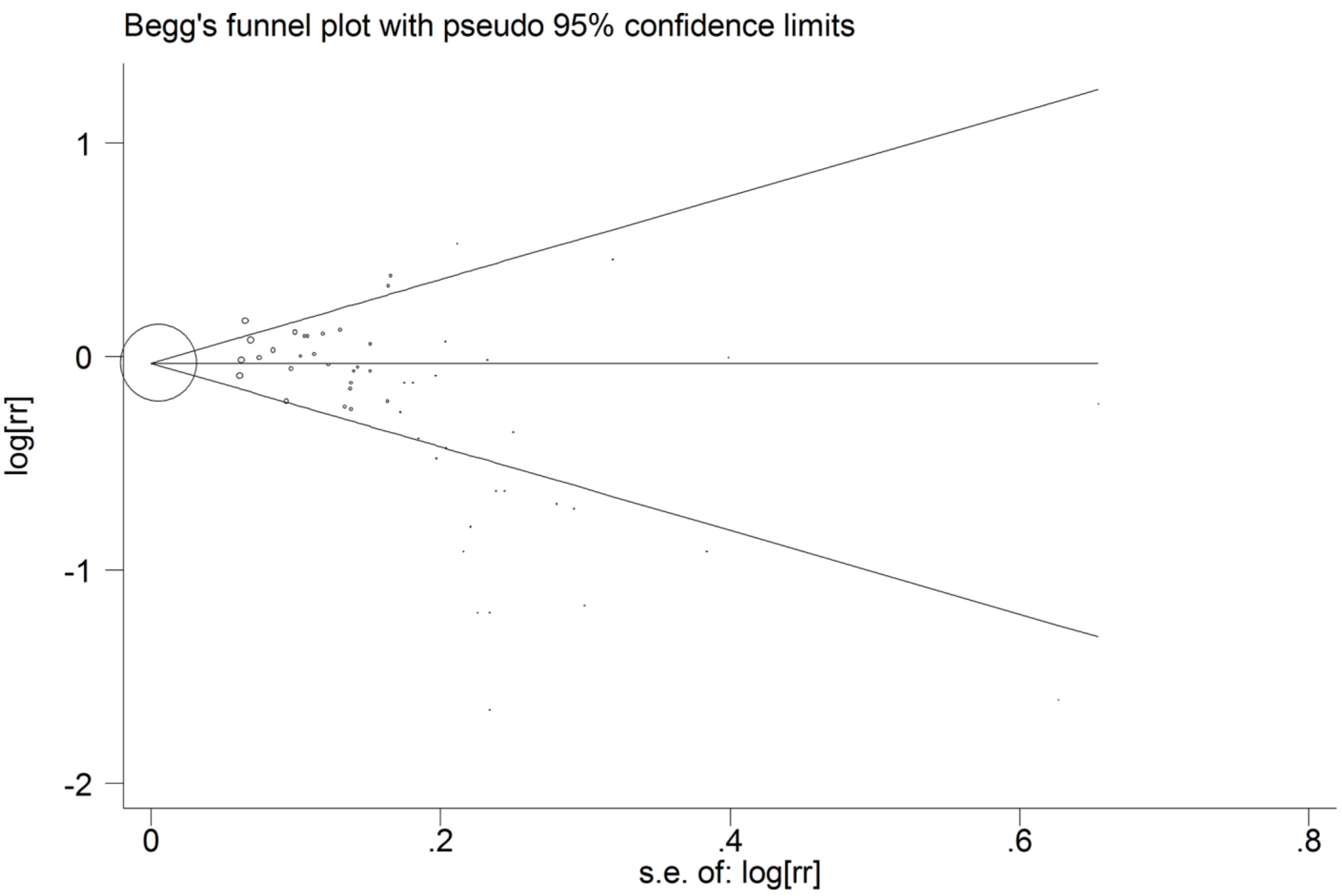

Supplementary Figure 1. Funnel plot of meta-analysis of breast cancer risk in relation to highest vs lowest categories of vitamin C intake.

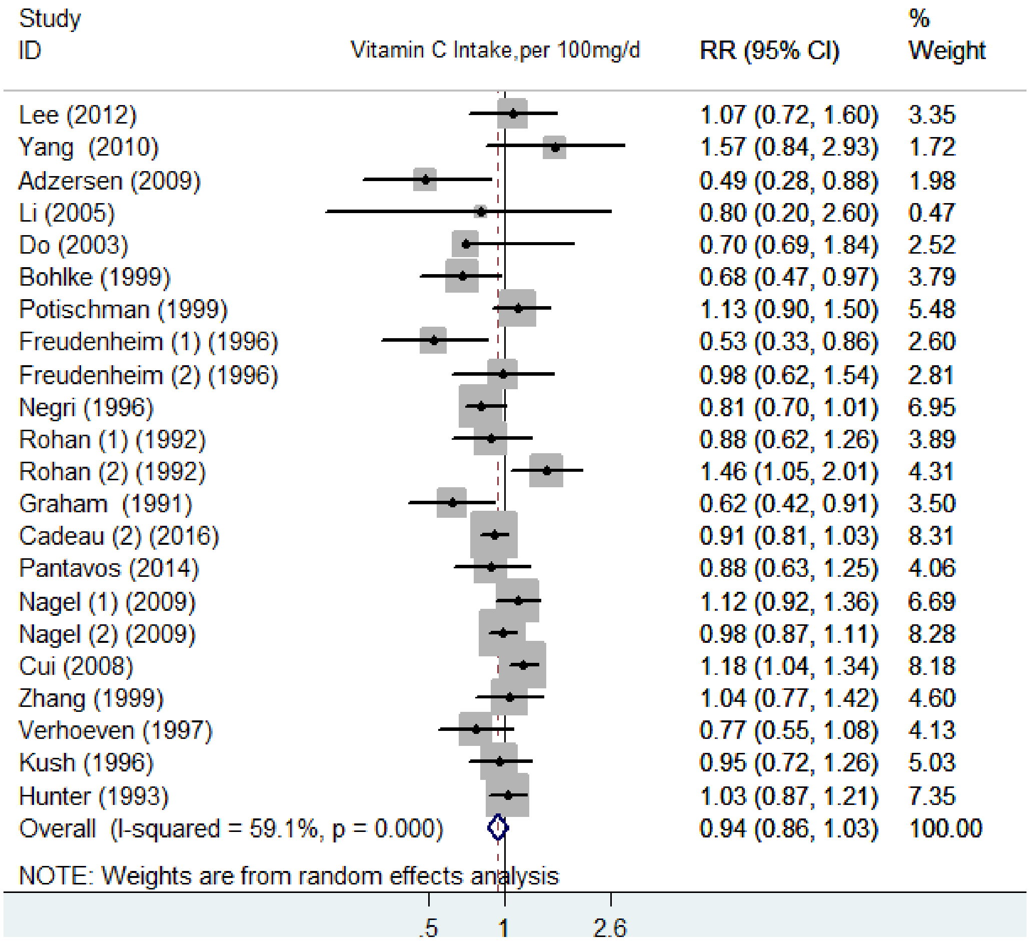

Supplementary Figure 2. Forest plot of meta-analysis of the association between vitamin C intake increment (per 100 mg/d) and breast cancer risk. Abbreviations: RR, relative risk; CI, confidence interval.

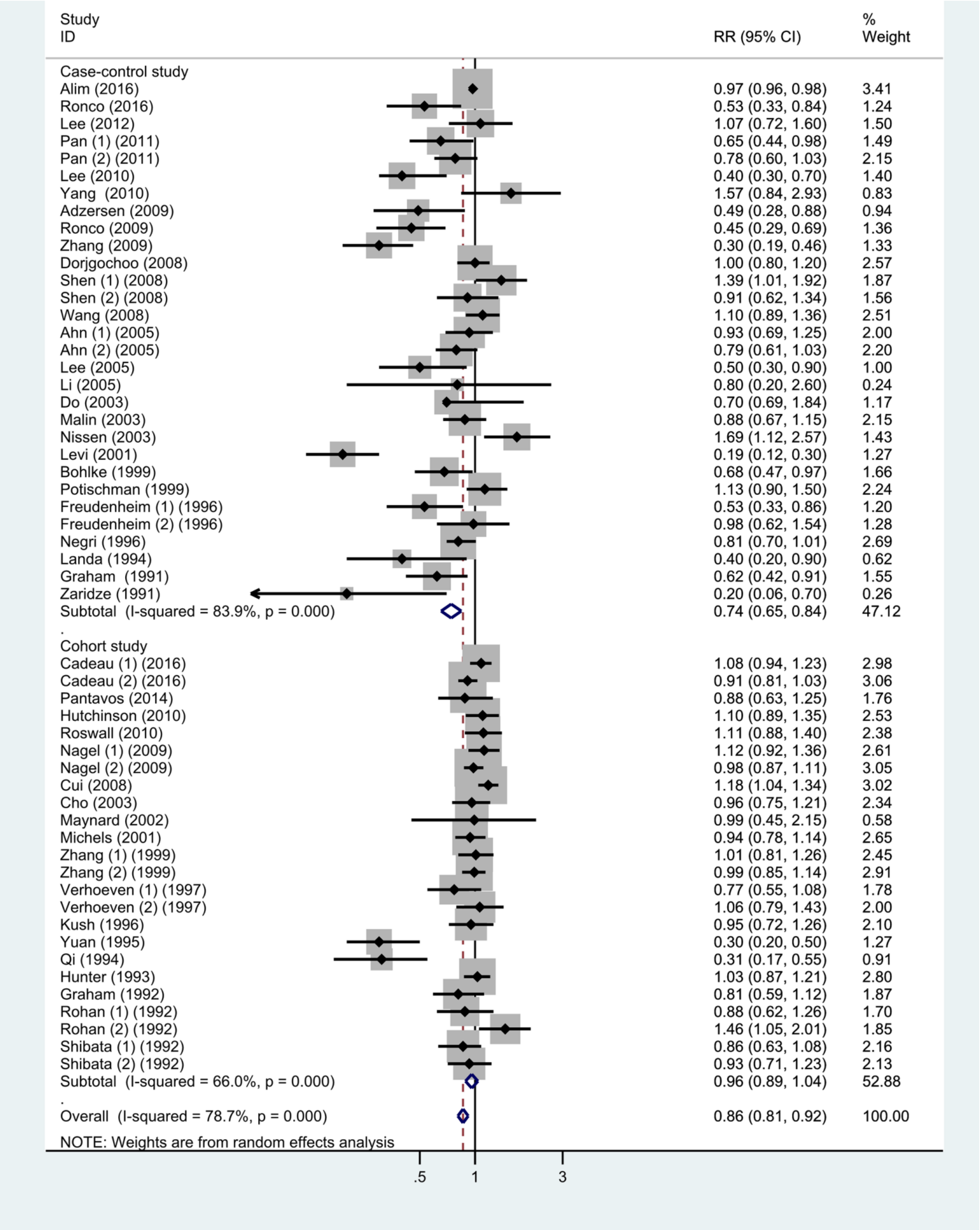

Supplementary Figure 3. Subgroup analyses of the associations between vitamin C intake and breast cancer risk stratified by study design. Abbreviations: RR, relative risk; CI, confidence interval.

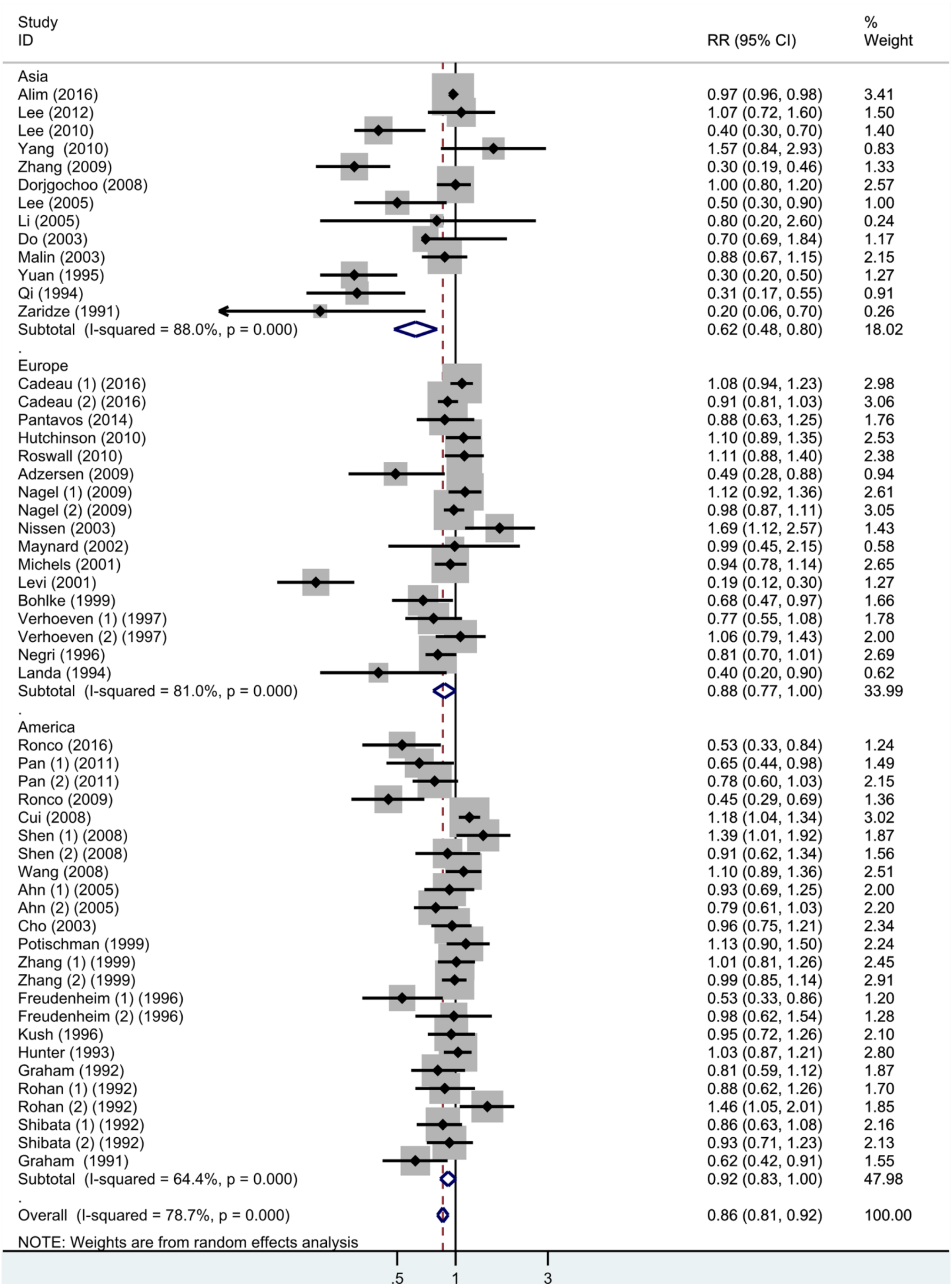

Supplementary Figure 4. Subgroup analyses of the associations between vitamin C intake and breast cancer risk stratified by geographic locations. Abbreviations: RR, relative risk; CI, confidence interval.

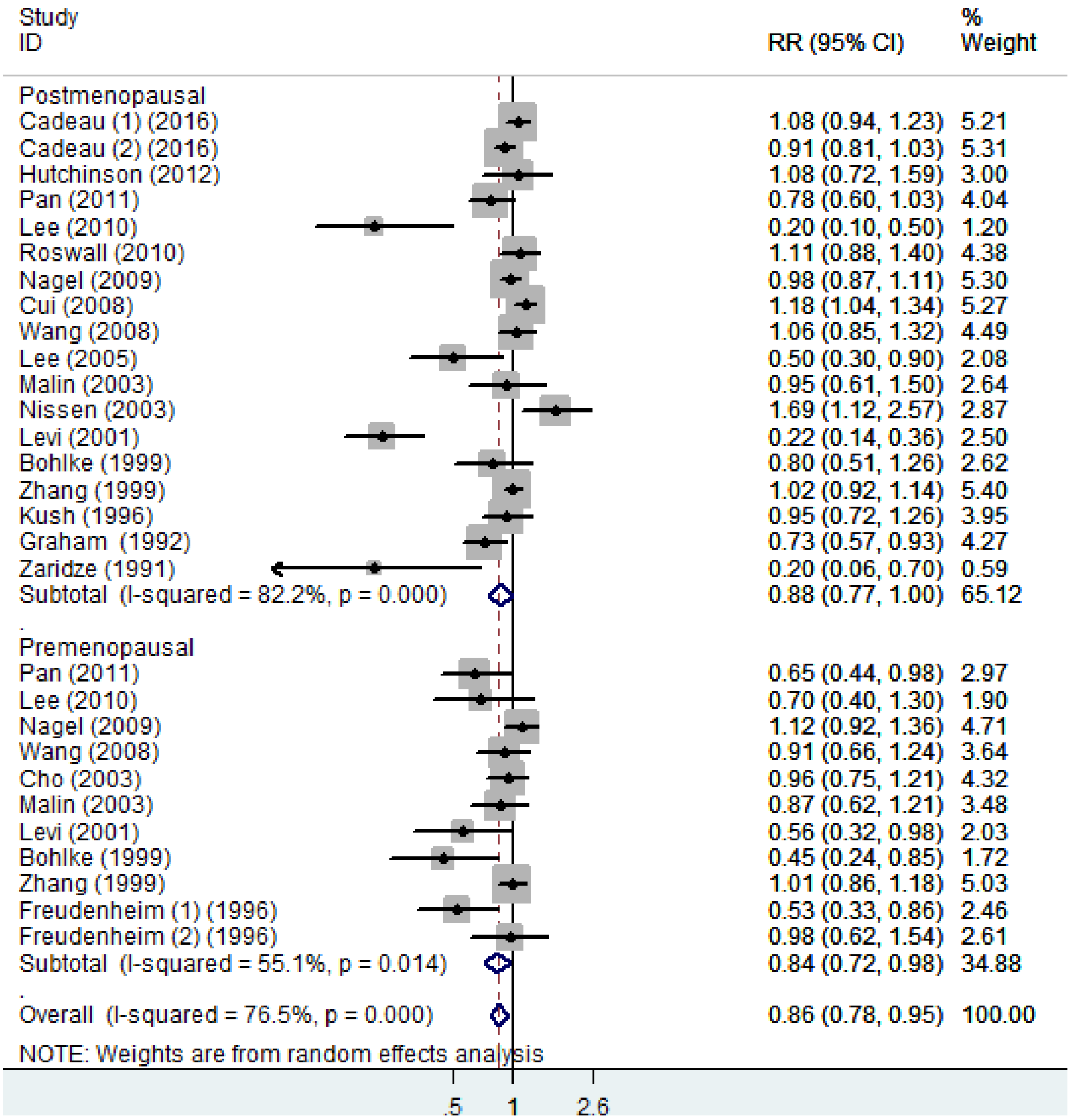

Supplementary Figure 5. Subgroup analyses of the associations between vitamin C intake and breast cancer risk stratified by menopausal status. Abbreviations: RR, relative risk; CI, confidence interval.
