# Supplementary material for: Association of vitamin C intake with breast cancer risk and mortality: a meta-analysis of observational studies
Source: Aging (Albany NY). 2020 Sep 29;12(18):18415–35. doi: 10.18632/aging.103769 (PMC7585084; doi:10.18632/aging.103769)
Supplement: Supplementary Table 1 [file aging-12-103769-s001..docx]

| **Supplementary Table 1. Characteristics of studies included in the meta-analysis of vitamin C intake and breast cancer risk.** | | | | | | | | |
| --- | --- | --- | --- | --- | --- | --- | --- | --- |
| **Author** | **Country** | **Study type** | **Follow-up period (year)** | **Age**  **(year)** | **No. of cases/controls/**  **persons** | **Vitamin C Intake**  **(mg/day)** | **Adjusted RR(95%CI)^a^** | **Adjustment factors** |
| Alim  2016 | Turkey | Case -  control | _ | Mean.51 | 40/40 | dietary intake vs no intake | 0.97 (0.96–0.98) | Age, BMI, menopausal status, educational Status, physical activity, occupation, current alcohol use, total energy intake |
| Cadeau  2016 | France | Cohort | 1995-2008 | 40–65 | 2482/57403 | dietary intake vs no intake  Supplement intake Highest / Lowest | 1.08 (0.94–1.23)  0.91 (0.81–1.03) | Age, BMI, menopausal status, smoking habit, drinking habit, physical activity, family history of breast cancer in a first degree  relative, age at menarche, parity, hormone use, total nonalcohol energy, and referral pattern |
| Ronco  2016 | USA | Case -  control | 1996-2004 | NA | 572/889 | dietary intake  Q4 vs Q1 | 0.53 (0.33–0.84) | Hospital, residence, age, menopausal status, family history of BC in 1st and 2nd degree, body mass index, dietary energy smoking status, alcohol drinking frequency and total red meat, vegetables, fruits, mate, tea and coffee |
| Pantavos  2014 | Netherlands | Cohort | 17 | Mean.67 | 199/46013 | dietary intake H / L | 0.88 (0.63–1.25) | Age, BMI, educational level, family history of breast cancer (yes or no), smoking status and alcohol consumption, use of multivitamin supplement (yes or no) |
| Lee  2012 | Koera | Case -  control | 2001-2002 | Mean.48 | 512/512 | Dietary intake  >210.3 vs ≤106.5 | 1.07 (0.72–1.60) | Age, body mass index, education, family history of breast cancer,  and age at first full-term pregnancy |
| Pan  2011 | Canada | Case -  control | 2004-2007 | 20-76 | 2362/2462 | Total H / L  Dietary intake H / L  Supplement intake H / L | 0.74 (0.59–0.92)  1.24 (1.08–1.42)  0.80 (0.61–1.04) | Age, province of residence, education, smoking pack years, alcohol consumption, BMI, recreational physical activity, number of live births, age at menarche, and total energy intake |
| Hutchinson  2010 | UK | Cohort | 4 | 37-79 | 523/12453 | Supplement yes/ no | 1.10 (0.89-1.35) | Age, BMI, social class, marital status, children, smoking status, level of physical activity, low alcohol consumption, red meat servings, total fruit and vegetable servings. |
| Lee  2010 | Korea | Case -  control | 2001-2003 | Mean.49 | 323/323 | Total ≥208.0 vs ≤105.4 | 0.40 (0.30–0.70) | Age, BMI, education, age of first full term pregnancy, family history of breast cancer, alcohol consumption, and pack year of cigarette smoking |
| Roswall  2010 | Denmark | Cohort | 10.6 | 50–64 | 1072/26224 | Total >203.2 vs ≤97.8  Dietary >145.2 vs ≤97.8  Supplement >64 vs ≤76.5 | 1.11 (0.88–1.40)  1.15 (0.92–1.44)  0.96 (0.77–1.21) | total intake micronutrients, alcohol intake, body mass index, hormone replacement therapy (HRT) use, duration of HRT use, number of births, parity, age at first birth and school education |
| Yang  2010 | Korea | Case -  control | 2004-2006 | 30-65 | 362/362 | Dietary Q5 vs Q1 | 1.51 (0.84–2.93) | Age, dietary fat), family history of breast cancer, and age at birth of first child, education (year), and alcohol intake |
| Adzersen  2009 | Germany | Case -  control | 1998-2000 | 25–75 | 310/353 | Dietary >134.4 vs <58.5 | 0.49 (0.28–0.88) | Age, total energy intake, age at menarche/ first birth/ menopause; mother/sister with breast cancer, current smoking, history of benign breast disease, BMI, consumption of alcohol et al |
| Nagel  2009 | Europe | Cohort | 8.8 | 35-70 | 7502/ 520000 | Dietary Q5 vs Q1 (pre-)  Dietary Q5 vs Q1 (post-) | 1.12 (0.92–1.36)  0.98 (0.87–0.11) | Age, energy from protein and carbohydrates, saturated fatty acids, monounsaturated fatty acids, polyunsaturated fatty acids, weight, height, age at menarche, parity, age at first pregnancy, use of hormone therapy at recruitment, smoking status, physical activity index, education |
| Ronco  2009 | Uruguay | Case -  control | 1994-1997 | 20-89 | 400/405 | Dietary Q4 vs Q1 | 0.45 (0.29–0.69) | Age, residence, urban/rural status, family history of breast cancer in a 1st-degree relative, body mass index, age at menarche, parity, menopausal status, and total energy intake. |
| Zhang  2009 | China | Case -  control | 2007-2008 | 25-70 | 438/438 | Dietary Q4 vs Q1 | 0.30 (0.19–0.46) | Age at menarche, BMI, history of benign breast disease, mother/sister/daughter with breast cancer, physical activity,  passive smoking and total energy intake |
| Cui  2008 | USA | Cohort | 7.6 | 50–79 | 508/84805 | Total >686 vs ≤97  Dietary >158 vs ≤67  Supplement >711 vs 0 | 1.18 (1.04–1.34)  1.06 (0.92–1.22)  1.16 (1.04–1.30) | energy intake, age at baseline, ethnicity, educational level, age at menarche, age at menopause, parity, age at first full-term pregnancy, oral contraceptive use, postmenopausal hormone use, BMI, physical activity, alcohol drinking, dietary folate intake, tobacco smoking, hysterectomy, bilateral oophorectomy, history of benign breast disease, and family history of breast cancer |
| Dorjgochoo  2008 | China | Case -  control | 2002-2005 | 20-70 | 3454/3474 | Supplement H / L | 1.00 (0.80–1.20) | Age (continuous), education, age at menarche categories), age at 1st birth (categories), livebirth, BMI (categories), menopausal status |
| Shen  2008 | USA | Case -  control | 1996-1997 | Mean.50 | 1026/1070 | Total Q4 vs Q1 (pre-)  Total Q4 vs Q1 (post-) | 1.39 (1.01–1.92)  0.91 (0.62–1.34) | Age at menarche, parity, lactation, history of fertility problems, body mass index at reference, BMI at age 20, first degree family history of breast cancer, history of benign breast disease, menopausal status, oral contraceptive use, hormone replacement use, smoking status, alcohol drinking, race, education, religion and marital status |
| Wang  2008 | USA | Case -  control | 1999-2004 | 25–79 | 1498/4850 | Total Q4 vs Q1 | 1.10 (0.89–1.36) | Age, education level, BMI, total METs per week for moderate and vigorous physical activity, total calorie intake per day, cigarette smoking, alcohol intake, and center |
| Ahn  2005 | USA | Case -  control | 1996-1997 | >20 | 1008/1056 | Total H / L (pre-)  Total H / L (post-) | 0.93 (0.69–1.25)  0.79 (0.61–1.03) | Age, family history, body mass index, and total calories, total energy intake |
| Lee  2005 | China | Case -  control | 1996-1999 | 25-74 | 418/349 | Total Q4 vs Q1 | 0.50 (0.30–0.90) | Age, education and total calories, age at menarche, menopause, age at first pregnancy, and parity |
| Li  2005 | China | Case -  control | 1995-2000 | >35 | 622/862 | Total >97.6 vs <55 | 0.80 (0.20–2.60) | Age, education, age at first live birth, menopause, years of oral contraceptive use, |
| Cho  2003 | USA | Cohort | 8 | Mean.36 | 714/90655 | Total Q5 vs Q1 | 0.96 (0.75–1.21) | Age, smoking, height, parity and age at first birth, body mass index, age at menarche, family history of breast cancer, history of benign breast disease, oral contraceptive use, menopausal status, alcohol intake, and animal fat |
| Do  2003 | Koera | Case -  control | 1999-2000 | 20-69 | 224/250 | Total >350.1 vs ≤151.01 | 0.70 (0.69–1.84) | Age at menarche, total menstrual periods, pregnancy, total number of full term delivery, total periods of breast feeding, family history of breast cancer and current BMI, total energy intake |
| Malin  2003 | China | Case -  control | 1996-1998 | Mean.47 | 1459/1556 | Dietary Q5 vs Q1 | 0.88 (0.67–1.15) | Age, menopausal status, reproductive history, hormone use, dietary habits, prior disease history, physical activity, tobacco and alcohol use, weight and family history of cancer |
| Nissen  2003 | Denmark | Case -  control | 1993-1997 | 50-64 | 418/394 | Total >300 vs ≤60 | 1.69 (1.12–2.57) | Age at first birth, history of benign breast disease surgery, school education, years of use of HRT, alcohol intake and BMI, total intake of the other two vitamins |
| Maynard  2002 | UK | Cohort | 8 | NA | 82/1959 | Dietary Q4 vs Q1 | 0.99 (0.45–2.15) | Age, and energy intake, BMI, family history of breast cancer |
| Michels  2001 | Sweden | Cohort | 1987-1990 | 40–76 | 1271/59036 | Dietary H vs L | 0.94 (0.78–1.14) | Age, family history of breast cancer, height, body mass index, education, parity, age at first birth, total caloric intake and intake of alcohol, fiber and monounsaturated fatty acids. |
| Levi  2001 | Sweden | Case -  control | 1993-1999 | 23-74 | 289/469 | Total H / L | 0.19 (0.12–0.30) | Age, education, parity, menopausal status, body mass index, total  energy intake, and alcohol drinking |
| Bohlke  1999 | Greece | Case -  control | 1989-1991 | 55.4 | 819/1548 | Total >343.1 vs ≤142.9 | 0.68 (0.47–0.97) | Age, birth place, body mass index, parity, age at first birth, age at menarche, menopausal status, and total energy intake |
| Potischman  1999 | USA | Case -  control | 1990-1992 | 20-44 | 568/1451 | Total >390 vs ≤95 | 1.13 (0.90–1.15) | Age at diagnosis, study site, ethnicity, education, age at first birth, alcohol intake, years of oral contraceptive use and smoking status |
| Zhang  1999 | USA | Cohort | 14 | 30–55 | 2697/83234 | Total Q5 vs Q1 (pre-)  Total Q5 vs Q1 (post-) | (0.81–1.26)  (0.85–1.14) | Age, length of follow-up, total energy intake, parity, age at first birth, age at menarche, history of breast cancer in mother or a sister, history of benign breast disease, alcohol intake, body mass index at age 18 years, weight change from age 18 years and height in inches. |
| Verhoeven  1997 | Netherlands | Cohort | 4.3 | 55-69 | 519/62537 | Dietary Q5 vs Q1  Supplement yes / no | 0.77 (0.55–1.08)  1.06 (0.79–1.43) | Age, energy intake, alcohol intake, history of benign breast disease, maternal breast cancer, breast cancer in sister(s), age at menarche, age at menopause, age at first birth, parity |
| Freudenheim  1996 | USA | Case -  control | 1986-1991 | ≥40 | 297/311  290/308 | Dietary >224 vs ≤131  Supplement >264 vs 0 | 0.53 (0.33–0.86)  0.98 (0.62–1.54) | Age. education, age at first birth, age at menarche, first-degree relative with breast cancer, previous benign breast disease, body mass index, and kilocalories by residuals |
| Kush  1996 | USA | Cohort | 2 | 55-69 | 879/41836 | Total >392 vs ≤112  Dietary >198 vs ≤87  Supplement >1000 vs 0 | 0.95 (0.72–1.26)  1.01 (0.69–1.48)  0.78 (0.47–1.30) | age, energy intake, age at menarche, age at menopause, age at first live birth, parity, body mass index at time of baseline questionnaire, family history of breast cancer, history of benign breast disease, alcohol intake, and educational attainment |
| Negri  1996 | Italia | Case -  control | 1991-1994 | Mean.55 | 2569/2558 | Total Q5 vs Q1 | 0.81 (0.70–1.00) | Age, education, parity, and intake of energy, alcohol, other nutrients (beta-carotene, vitamin E, riboflavin, calcium, potassium) |
| Yuan  1995 | China | Case -  control | 1963-1978 | 20-69 | 834/834 | Dietary H vs L | 0.30 (0.20–0.50) | Age, education, age at menarche , total energy intake |
| Landa  1994 | Spain | Case -  control | 1988-1991 | Mean.59.5 | 100/100 | Dietary Q3 vs Q1 | 0.40 (0.20–0.90) | Age, education, parity, menopausal status, body mass index, total  energy intake, and alcohol use |
| Qi  1994 | China | Case -  control | 1986-1987 | NA | 2616/2316 | Total >140 vs ≤100 | 0.31 (0.17-0.55) | Age, education, age at menarche, age at menopause and age at first birth, total calories, average family income, cigarette smoking and alcohol consumption, history of malignant tumor |
| Hunter  1993 | USA | Cohort | 4 | 34-59 | 666/89494 | Supplement >359 vs <93 | 1.03 (0.87–1.12) | Age, BMI, education, length of follow-up, energy intake, parity, age at birth, alcohol use, |
| Graham  1992 | USA | Cohort | 7 | Mean.45 | 344/17401 | Dietary Q5 vs Q1 | 0.81 (0.59–1.12) | Age, education |
| Rohan  1992 | Canada | Case -  control | 1982-1987 | 40-59 | 519/1182 | Dietary Q5 vs Q1  Supplement Q3 vs Q1 | 0.88 (0.62–1.26)  1.46 (1.05–2.01) | Age, energy intake, age at menarche, surgical menopause, age at first livebirth, years of education, family history of breast  cancer, and history of benign breast disease |
| Shibata  1992 | USA | Cohort | 8 | Mean.43.8 | 219/45941 | Dietary H vs L  Supplement yes vs no | 0.86 (0.63–1.08)  0.93 (0.71–1.23) | Age, BMI, education, length of follow-up, energy intake, parity, age at menarche, age at birth, alcohol use, a history of benign breast disease |
| Graham  1991 | USA | Case -  control | NA | 41-85 | 439/434 | Total >229 vs <128 | 0.62 (0.42–0.97) | Age, education, age at first pregnancy, age at menarche, relative with breast cancer, nutritional traits |
| Zaridze  1991 | Russia | Case -  control | 1987-1989 | NA | 85/81 | Total Q4 vs Q1 | 0.20 (0.06–0.70) | Age, BMI, menopausal status, educational Status, physical activity, occupation, nationality ,total energy intake |

Abbreviations: RR, relative risk; CI=confidence interval; Ref, reference; NO. of cases/subjects, number of cases/subjects; BMI=body mass index (kg/m^2); HRT=hormone replacement therapy; Post- =postmenopausal; Pre- =premenopausal.

a. The RRs of most studies used the lowest category of vitamin C intake levels as a reference in the meta-analysis
