# Supplementary material for: Association of vitamin C intake with breast cancer risk and mortality: a meta-analysis of observational studies
Source: Aging (Albany NY). 2020 Sep 29;12(18):18415–35. doi: 10.18632/aging.103769 (PMC7585084; doi:10.18632/aging.103769)
Supplement: Supplementary Table 2 [file aging-12-103769-s002..pdf]

SUPPLEMENTARY TABLE

Supplementary Table 2. Characteristics of studies included in the meta-analysis of vitamin C intake and breast cancer survival.

Author	Country Study type	Follow-up period (year)	Age (year)	No. of cases/deaths/ recurrence	Estimation target	Vitamin C Intake (mg/day)	Adjusted HR(95%CI)[a]	Adjustment factors
Harris 2013	Sweden Cohort	7.8	Mean 65	3405/1055<br>3405/416	Death From All Causes<br>Death From Breast Cancer	>92.5 vs <42.9<br>Highest / Lowest	0.84 (0.71–1.00)<br>0.75 (0.57–0.99)	Age, energy intake, education level, marital status, menopausal status at diagnosis, body mass index, alcohol intake and calendar year of diagnosis.
Poole 2013	USA Cohort	5	Mean.54.8	10222/659<br>10222/955	Death From Breast Cancer<br>Death From All Causes	Highest/Lowest<br>Highest / Lowest	0.87 (0.73–1.03)<br>0.82 (0.71–0.95)	Age at diagnosis, exercise, stage, treatment, BMI, smoking, menopausal status, race
Greenlee 2012	USA Cohort	10	18-79	2264/214<br>2264/393<br>2264/375	Death From Breast Cancer<br>Death From All Causes<br>Breast Cancer Recurrence	Highest/Lowest<br>Highest / Lowest<br>Highest/Lowest	0.71 (0.54–0.92)<br>0.78 (0.61–1.01)<br>0.82 (0.58–1.16)	Age at diagnosis, race/ethnicity, education, breast cancer stage at diagnosis, number positive lymph nodes, tumor hormone receptor status, chemotherapy received, radiation therapy received, hormone therapy received, body mass index 1 year before diagnosis, smoking history at enrollment, alcohol consumption at enrollment, physical activity at enrollment, daily servings of fruits and vegetables at enrollment, and comorbidity score at enrollment
Nechuta 2011	China Cohort	4.1	20-75	4877/444<br>4877/532	Death From All Causes<br>Breast Cancer Recurrence	Highest / Lowest	0.81 (0.61–1.07)	Age, ER/PR status, TNM stage, chemotherapy, radiotherapy, tamoxifen use, education, income, BMI, regular tea consumption, regular exercise participation, daily cruciferous vegetable intake, daily soy protein intake, and other vitamin variables
Saquib 2011	USA Cohort	9	53	3081/412	Death From All Causes	Highest/Lowest	1.1 (0.79–1.6)	Age at randomization, tumor stage, tumor grade, time since diagnosis, BMI, smoking, randomization group, hot flashes, group by hot flash interaction, and physical health
Rohan 2009	Australia Case - control	-	20-74	412/412	Death From Breast Cancer	>234 vs ≤71	0.74 (0.42–1.30)	Age at menarche, Quetelet index, age at 1st live birth, energy, education, history of benign breast disease, menopausal status, height, weight, ER,PR status
McEligot 2006	USA Cohort	1	Mean.65	516/96	Death From All Causes	Q3 vs Q1	0.45 (0.25–0.78)	Age at diagnosis, stage of disease, body mass index, parity, hormone replacement therapy use, alcohol use, multivitamin use, and energy intake, micronutrients

Maynard 2002	UK Cohort	8	NA	101/36	Death From Breast Cancer	Highest/Lowest	0.58 (0.19–1.84)	Age, and energy intake, BMI, family history of breast cancer, smoking status
Holmes 1999	USA Cohort	13	Mean.54	1982/378	Death From All Causes	Q5 vs Q1	0.80 (0.58–1.10)	Age, diet interval, calendar year of diagnosis, body mass index, oral contraceptive use, menopausal status, postmenopausal hormone use, smoking, age at first birth and parity, number of metastatic lymph nodes, and tumor size
Jain 1994	Canada Cohort	5	40-59	673/76	Death From Breast Cancer	>201.3 vs ≤11.07	0.43 (0.21–0.86)	Total energy, age at diagnosis, smoking, and body weight

Abbreviations: HR, hazard risk; CI=confidence interval; Ref, reference; NO. of cases/deaths/recurrence, number of cases/deaths/recurrence; BMI=body mass index (kg/m^2); ER, estrogen receptor; PR, progesterone receptor; TNM, tumor, Node, Metastasis.

[a]The HRs of all studies used the lowest category of vitamin C intake levels as a reference in the meta-analysis.
